# Supplementary material for: Porphyromonas gingivalis Gingipains Destroy the Vascular Barrier and Reduce CD99 and CD99L2 Expression To Regulate Transendothelial Migration
Source: Microbiol Spectr. 2023 May 18;11(3):e04769-22. doi: 10.1128/spectrum.04769-22 (PMC10269447; doi:10.1128/spectrum.04769-22)
Supplement: Supplemental file 1 — Supplemental material. Download spectrum.04769-22-s0001.pdf, PDF file, 0.6 MB [file spectrum.04769-22-s0001.pdf]

Supplementary File

Porphyromonas gingivalis gingipains destroy the vascular barrier and reduce CD99 and CD99L2 expression to regulate transendothelial migration

Zhaolei Zou[1#], Juan Fang[1#], Wanting Ma[1#], Junyi Guo[1], Zhongyan Shan[1], Da Ma[1], Qiannan Hu[1], Liling Wen[1], Zhi Wang[1*]

# Zhaolei Zou, Juan Fang and Wanting Ma contributed equally to this work.

[1]Hospital of Stomatology, Guanghua School of Stomatology, Guangdong Provincial Key Laboratory of Stomatology, Sun Yat-Sen University, Guangzhou, Guangdong, PRC.

Corresponding Author:

Zhi Wang, Hospital of stomatology, Guanghua School of Stomatology, Guangdong Provincial Key Laboratory of Stomatology, Sun Yat-Sen University, No. 56, Lingyuanwest Road, Guangzhou, Guangdong 510055, P. R. China.

E-mail: wangzh75@mail.sysu.edu.cn

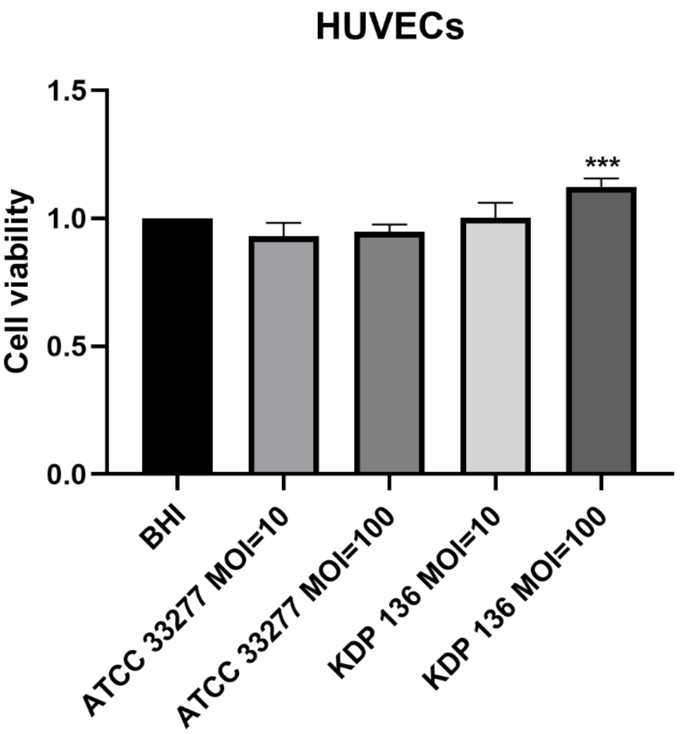

Supplementary figure S1. CCK8 assay for cell viability of HUVECs after 24h infection with *P. gingivalis* ATCC 33277 and KDP 136. Data are the means ± SD from three independent experiments. ****P*<0.001 compared to BHI group.

a

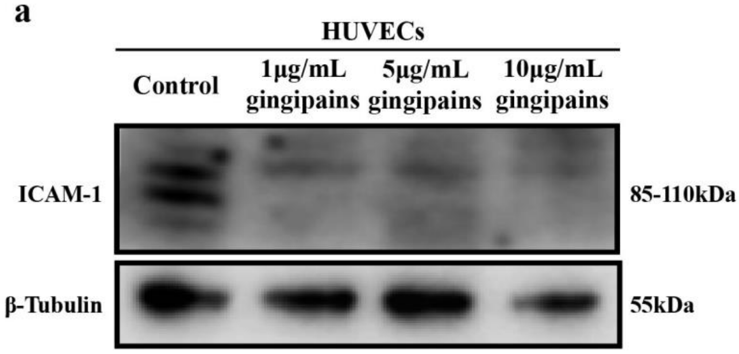

Supplementary figure S2. ***P. gingivalis*** **gingipains reduces ICAM-1 expression on HUVECs.** (a) HUVECs were incubated with indicated concentrations of gingipains for 24h and the expression of ICAM-1 was detected using western blots.

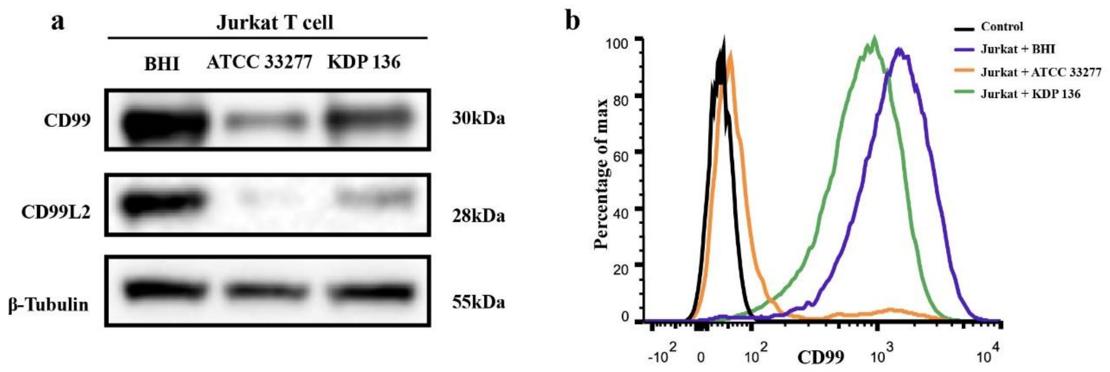

Supplementary figure S3. ***P. gingivalis*** **reduces CD99 and CD99L2 expression on Jurkat cells.** Jurkat cells were co-cultured with BHI, ATCC 33277 or KDP 136 for 24 h. (a) Western blotting to examine the expression of CD99 and CD99L2 expression on Jurkat. (b) Flow cytometry results showing the expression of CD99 on Jurkat co-cultured with BHI, ATCC 33277 or KDP 136.

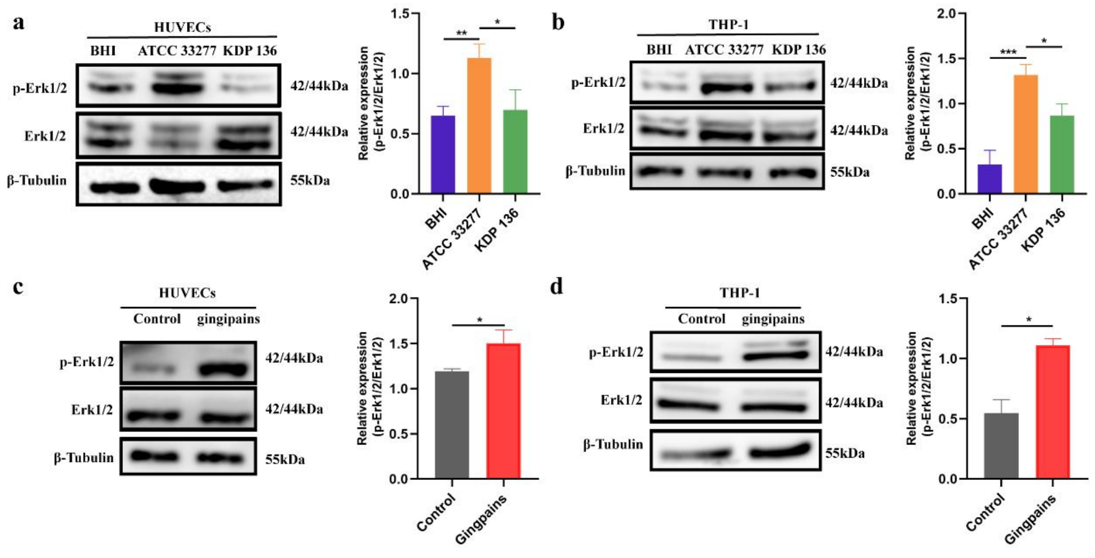

Supplementary figure S4. *P. gingivalis* specifically activates the Erk1/2 pathway through gingipains. (a-b) Western blotting to examine the expression of p-Erk1/2 and Erk1/2 in HUVECs (a) and THP-1 (b) at 24h after co-cultured with BHI, ATCC 33277 or KDP 136. (c-d) HUVECs (c) or THP-1 (d) were co-cultured with 10 μg/mL gingipains, western blots were performed to examine the expression of p-Erk1/2 and Erk1/2. Data are the means ± SD from at least three independent experiments. *P<0.05, **P<0.01, ***P<0.001.

Materials and methods

Cell culture

Immortalized human umbilical vein endothelial cells (HUVECs), human THP-1 cells and Jurkat were purchased from ATCC. HUVECs were cultured in DMEM supplemented with 10% heat-inactivated FBS at 37°C and 5% CO_2. THP-1 and Jurkat were cultured in RPMI-1640 supplemented with 10% heat-inactivated FBS at 37°C and 5% CO_2.

Bacterial culture and preparation of *P. gingivalis* supernatant

P. gingivalis ATCC 33277 and *E. coli* ATCC 25922 were purchased from ATCC. Gingipains-deficient *P. gingivalis* KDP 136 (ΔrgpA ΔrgpB Δkgp)[1] were kindly provided by Dr. Jinlong Gao from Faculty of Medicine and Health, University of Sydney. *P. gingivalis* were grown in BHI broth medium supplemented with vitamin K1 (0.5 μg/ml) and hemin (5 μg/ml) in an anaerobic system (oxygen concentration < 0.16%). *E. coli* were cultured in LB broth at 37°C. To prepare for *P. gingivalis* supernatant, *P. gingivalis* ATCC 33277 was grown in BHI medium. Culture supernatant was collected after centrifugation (6000 × g, 4°C, 10 min) and used for following experiment.

P. gingivalis gingipains extraction and purification

P. gingivalis gingipains were extracted and purified as previously described[2,3]. Briefly, *P. gingivalis* cultures were centrifuged (12,000 × g, 45 min, 4°C) and filtered through a

0.45μm filter (Millipore). The extracellular cultures were precipitated at -20°C in a 60:40 ratio of acetone to cell-free medium. Then the precipitates were centrifuged (12,000 × g, 30 min, 4°C) and resuspended in a solution containing 150 mM NaCl, 20 mM Bis-Tris and 5 mM $CaCl_2$. After dialysis, the sample was centrifuged (34,000 × g, 1 h, 4°C), and the resulting supernatant was concentrated in a pressurized stirring concentrator (Millipore) with a 10,000-molecular-weight-cutoff membrane at 4°C[3]. The gingipains extract was clarified by centrifugation (192,000 × g, 1 h, 4°C), and stored at -80°C. Gingipains activity was tested as previously described[3] and identified using SDS-PAGE[2,4]. The concentrations of gingipains were determined by BCA for subsequent experiments.

Fluorescence permeability assay

Costar transwell inserts (6.5 mm, 0.4 μm; Corning) were fibronectin-coated (10 μg/ml) for 30 min at 37 °C before seeding HUVECs that were cultured until confluent. HUVECs were incubated with BHI, *P. gingivalis* supernatant, ATCC 33277 and KDP 136 (Multiplicity of infection, MOI = 100) for 24 h. FITC-dextran (Sigma-Aldrich, 46945) was added to the upper chamber (1 mg/mL). At the indicated times, 20 μL medium were removed from the lower chamber and diluted 1:20. Fluorescence was measured in triplicate in a microplate reader. Each experiment was performed in duplicate and repeated at least three times. For the effect of gingipains, HUVECs were incubated with the indicated concentrations of gingipains for 24 h and the permeability assay described above were performed.

***E. coli* transwell penetration test**

HUVECs were cultured in fibronectin-coated transwell inserts as described above. *P. gingivalis* ATCC 33277 or KDP 136 were added to HUVECs at an MOI of 100:1 and *E. coli* was added at an MOI of 100:1. After 1 h incubation at 37°C, aliquots were taken from the lower chamber, diluted by a certain factor, and plated on LB agar plates, then incubated overnight at 37°C in air. The colony forming units (CFU) numbers of *E. coli* were counted.

Cell Counting Kit-8 Assay

3*10^4 HUVECs were seeded in a 96-well plate, infected with ATCC 33277 and KDP 136 at different MOIs for 24 h. The medium was removed, washed with PBS, and then filled with CCK-8 (DOJINDO, CK04) solution. After incubation at 37 °C for 1h, the absorbance at 450 nm was detected with a microplate reader.

Western Blot

For in-vitro experiments, cells were co-cultured with BHI, ATCC 33277, KDP 136 (MOI=100) or indicated gingipains for 24h. Using RIPA Lysis Buffer (CWBIO, CW2333) containing protease and phosphatase inhibitor cocktail (CWBIO) to extract protein. An equal amount of protein (25 μg) from each sample was subjected to 4-12% SDS-PAGE and then transferred onto 0.45 μm polyvinylidenedifluoride (PVDF) membranes (Millipore). The membranes then were blocked with 5% skimmed milk and

incubated with primary antibodies at 4°C overnight followed by incubation with HRP-conjugated secondary antibodies for 1h. The blots were visualized using GeneGnome XRQ and quantified by ImageJ. The used primary antibodies were listed as follows.

Antibodies used for western blots

Antibody	Source	Dilution ratio	Clone
PECAM-1	CST	1:1000	3528S
VE-cadherin	CST	1:1000	2500S
ICAM-1	Wanleibio	1:1000	WL02268
Integrin αM	CST	1:1000	49420S
Integrin β2	Wanleibio	1:1000	WL02673
CD99	HUABIO	1:1000	ER1803-81
CD99L2	Abclonal	1:1000	A15907
p-Akt (Ser473)	CST	1:2000	4060S
Akt (pan)	CST	1:1000	4685S
p-Erk1/2	CST	1:2000	4370S
Erk1/2	CST	1:1000	4695S
β-Tubulin	Earthox	1:4000	E021040-02
β-actin	CST	1:1000	4970S

RNA extraction and real time-qPCR (RT-qPCR)

Total RNAs extracted using RNA-Quick Purification Kit (ESscience, RN001). The RNAs (1μg) were then concerted into cDNA using PrimeScript RT Master Mix (Takara, RR036A). AceQ® Universal SYBR® qPCR Master Mix (Vazyme, Q511-02) were used to perform RT-qPCR. After normalization to GAPDH expression, the expression of the target gene was quantified by the 2-ΔΔCt method. The primers to PECAM-1, VE-

cadherin, CD99, CD99L2, GAPDH were listed as follows.

Sequences of primers used for RT-qPCR.

Gene	Primer sequence (5’-3’)	Reference
PECAM-1	Forward: AAGATAGCCTCAAAGTCG	
	Reverse: CTGGGCATCATAAGAAAT	
VE-cadherin	Forward: GGCAAGATCAAGTCAAGCGTG	
	Reverse: ACGTCTCCTGTCTCTGCATGC	
CD99	Forward: CACCGAACCCACCCAA	
	Reverse: TCTCCATGTCCACCTCCC	
CD99L2	Forward: TCGGCGTTCCTTGTCTG	
	Reverse: GCCATCACCTCTTCCTCCT	
GAPDH (human)	Forward: AATCCCATCACCATCTTCC	
	Reverse: GAGTCCTTCCACGATACCAA	
Total bacteria	Forward: ACTCCTACGGGAGGCAGCAGT	Bücker [5]
	Reverse: ATTACCGCGGCTGCTGGC	
P. gingivalis	Forward: GTGAGGTAACGGCTCACCAA	Wu[6]
	Reverse: GTATCGCCCGTTATTCCCGT	
GAPDH (Mus musculus)	Forward: TGTTTCCTCGTCCCGTAG	
	Reverse: CAATCTCCACTTTGCCACT	

Immunofluorescence

HUVECs and THP-1 cells used for fluorescent staining of PECAM-1 and CD99 were inoculated onto slides, fixed with 4% paraformaldehyde for 20 min after experimental treatment and blocked with 5% BSA for 30 min, incubated with PECAM-1 (CST, 3528S) or CD99 (HUABIO, ER1803-81) antibodies at 4°C overnight. After adding

Alexa Fluor 488 or Alexa Fluor 594 conjugated secondary antibodies, aqueous blockers were used to cover the coverslips. Slices were visualized using a fluorescence microscope (Olympus FV3000).

Flow cytometry

To detect the expression of PECAM-1 and CD99 on cells surface after experimental treatment, cells were scraped and incubated with PECAM-1, CD99 antibodies (1:50) or a matching isotype control antibody for 1h at 4°C. After washing with PBS, cells were labelled with Alexa Fluor 488 conjugated antibody for 50 minutes.

To detect the expression of Integrin αM (CD11b) and Integrin β2 (CD18) on THP-1. Cells were incubated with FITC Anti-Human/Mouse CD11b (TONBO biosciences, 35-0112) and FITC Anti-Human CD18 (Biolegend, 302105) for 1h at 4°C. All cells were analyzed using BD LSRFFortessa (BD Biosciences).

Adhesion of THP-1 assay

HUVECs were cultured in 24 well plates until confluent. As described in previous study[7], for Fig. 3a, HUVECs were co-cultured with *P. gingivalis* for 24 h, and 1*10^5 Calcein-AM (Solarbio, IC4630) labeled THP-1 cells were added for 2 h; For Fig. 3c, THP-1 were co-cultured with *P. gingivalis* for 24 h, and labeled with Calcein-AM. Then 1*10^5 THP-1 cells were added to HUVECs. Un-adherent THP-1 cells were gently washed with PBS, imaged with fluorescence microscope (Zeiss), five fields of view were taken each time to calculate the amount of adherent THP-1, and analyzed by

ImageJ.

Transendothelial migration of THP-1 assay

Migration assays were performed in 24 well plate using fibronectin-coated Costar transwell inserts (6.5 mm, 8 μm; Corning) seeding HUVECs that were cultured until confluent. HUVECs were infected with *P. gingivalis* ATCC 33277 or KDP 136 (MOI=100) for 24 h. After washing with PBS, 100 μL of 2*10^6/mL THP-1 was added to the upper chamber. The lower chamber was filled with 500 μL medium plus 10 ng/mL of MCP-1 (Novoprotein, CM78). After 3 h, aspirate the lower chamber medium and count with a Beckman cell counter and calculate the portion of TEM THP-1 cells.

Mice and *P. gingivalis* tail vein injection model

Six-week-old male C57BL/6 mice were purchased from Beijing Vital River Laboratory Animal Technology Co., Ltd. All mice were maintained in a specific pathogen-free environment. All animal study protocols were performed under the guidelines of the Institutional Animal Care and Use Committee of Sun Yat-Sen University (SYSU-IACUC-2022-001542). After two weeks of co-house, mice were divided into two groups. Sham group was injected with 200 μL PBS through the tail vein, and *P. gingivalis* group was injected with ATCC 33277 resuspended in PBS (1*10^8 CFU/mL, 200 μL) every 3 days.

Evan's blue assay for in vivo vascular permeability

After 4 weeks, 200 μL of 0.5% Evan's blue (Solarbio, G1810) was tail vein injected, and the mice were sacrificed 30 minutes later. The organs were collected and thoroughly rinsed with PBS. Part of the tissue was cut and weighed, placed in 200 μL deionized formamide (YuanYe, R21111) and incubated at 60°C for 24 h. After centrifugation, 50 μL was drawn into a 96-well plate in triplicate, and the absorbance at 610 nm was measured by a microplate reader. Tissue penetrating dye amounts were expressed as absorbance/tissue weight.

Detection of total bacteria and *P. gingivalis* in mouse tissues

Tissue DNA Kit (Omega, D3396-02) was used to extract mice tissue DNA. 200μg DNA were used to perform RT-qPCR, methods and analyses were performed as described previously[5,6]. Total bacteria and *P. gingivalis* gene levels were normalized to GAPDH. The primers to total bacteria, *P. gingivalis* and GAPDH were listed in Table S2.

Immunofluorescence of vascular tissue

Mice lung tissues were washed in buffer and frozen in a cryostat at optimal cutting temperature compound. Cut into 5 μm sections and incubate with PECAM-1 (Abcam, ab281583, 1:50) antibody overnight at 4°C. After incubation with Alexa Flour 488 secondary antibody, aqueous blockers were used to cover the coverslips. Images were taken with Olympus fluorescence microscope and the fluorescence intensity of vascular PECAM-1 was analyzed by ImageJ.

H&E staining and immunohistochemistry (IHC)

Mice organs were harvested and fixed in 10% formalin for 24 h, then sectioned into 4 μm slices. The slices were then stained with hematoxylin and eosin (H&E). IHC was carried out in the following steps: dewaxing, rehydrated in graded alcohol, 3% hydrogen peroxide closure, Tris-EDTA (pH=8.0) high temperature antigen repair, antigen closure. Next slices were incubated with PECAM-1 (Abcam, ab281583, 1:4000) and CD99 (HUABIO, ER1803-81, 1:1000) antibodies respectively overnight at 4°C. After treatment with HRP-conjugated goat anti-rabbit secondary antibody, slides were stained with 3,3-diaminobenzidine (DAB). The stained slides were then scanned using an Aperio AT2 scanner (Leica). At least three random regions of interest (containing blood vessels) from each section were selected for quantification. Average PECAM-1 and CD99 H-scores were quantified by background subtraction using the Aperio eSlide Manager quantification software[8]. The threshold for scanning of different positive cells was set according to the standard control slices provided by Aperio[9].

Statistical analysis

All statistical analyses were implemented with GraphPad Prism 8.0. Unpaired two-tailed Student's t-test for two groups was used. Analysis of variance (ANOVA) was performed on multiple groups, followed by Dunnett's multiple comparison test or Tukey's multiple comparison test. Results are expressed as mean ± standard deviation (SD) from at least three independent experiments. $^*P<0.05$ was considered statistically significant.

Reference:

1 Shi, Y. *et al.* Genetic analyses of proteolysis, hemoglobin binding, and hemagglutination of Porphyromonas gingivalis. Construction of mutants with a combination of rgpA, rgpB, kgp, and hagA. *J Biol Chem* **274**, 17955-17960 (1999).

2 Zhang, F. *et al.* Signal-Regulated Protein Kinases/Protein Kinase B-p53-BH3-Interacting Domain Death Agonist Pathway Regulates Gingipain-Induced Apoptosis in Osteoblasts. *J Periodontol* **88**, e200-e210 (2017). https://doi.org:10.1902/jop.2017.160806

3 Sheets, S. M., Potempa, J., Travis, J., Casiano, C. A. & Fletcher, H. M. Gingipains from Porphyromonas gingivalis W83 induce cell adhesion molecule cleavage and apoptosis in endothelial cells. *Infect Immun* **73**, 1543-1552 (2005).

4 Chen, Y.-t., Song, X.-c., Zhang, F.-p. & Liang, M. [Expression of Bim, Bax and Bak in the process of gingipain-induced osteoblast apoptosis]. *Zhonghua Kou Qiang Yi Xue Za Zhi* **48**, 272-277 (2013). https://doi.org:10.3760/cma.j.issn.1002-0098.2013.05.005

5 Bücker, R. *et al.* α-Haemolysin of Escherichia coli in IBD: a potentiator of inflammatory activity in the colon. *Gut* **63**, 1893-1901 (2014). https://doi.org:10.1136/gutjnl-2013-306099

6 Wu, J.-S. *et al.* Porphyromonas gingivalis Promotes 4-Nitroquinoline-1-Oxide-Induced Oral Carcinogenesis With an Alteration of Fatty Acid Metabolism. *Front Microbiol* **9**, 2081 (2018). https://doi.org:10.3389/fmicb.2018.02081

7 Li, Q. *et al.* LOX-1 Regulates Porphyromonas gingivalis-Induced Monocyte Migration and Adhesion to Human Umbilical Vein Endothelial Cells. *Front Cell Dev Biol* **8**, 596 (2020). https://doi.org:10.3389/fcell.2020.00596

8 Wang, D. *et al.* A comprehensive profile of TCF1 progenitor and TCF1 terminally exhausted PD-1CD8 T cells in head and neck squamous cell carcinoma: implications for prognosis and immunotherapy. *Int J Oral Sci* **14**, 8 (2022). https://doi.org:10.1038/s41368-022-00160-w

9 Mao, L. *et al.* γ-Secretase inhibitor reduces immunosuppressive cells and enhances tumour immunity in head and neck squamous cell carcinoma. *Int J Cancer* **142** (2018). https://doi.org:10.1002/ijc.31115
